# Mapracorat, a novel selective glucocorticoid receptor agonist, inhibits hyperosmolar-induced cytokine release and MAPK pathways in human corneal epithelial cells

**Published:** 2010-09-02

**Authors:** Megan E. Cavet, Karen L. Harrington, Keith W. Ward, Jin-Zhong Zhang

**Affiliations:** Pharmaceutical R&D, Bausch & Lomb, Inc., Rochester, NY

## Abstract

**Purpose:**

Increasing evidence suggests that tear hyperosmolarity is a central mechanism causing ocular surface inflammation and damage in dry eye disease. Mapracorat (BOL-303242-X) is a novel glucocorticoid receptor agonist currently under clinical evaluation for use in the treatment of dry eye disease. This study assessed the anti-inflammatory effects of mapracorat in an in vitro osmotic stress model which mimics some of the pathophysiological changes seen in dry eye.

**Methods:**

Human corneal epithelial cells were cultured in normal osmolar media (317 mOsM) or 440 mOsM hyperosmolar media for 24 h. Luminex technology was used to determine the effect of mapracorat on hyperosmolar-induced cytokine release. Effects of mapracorat on mitogen-activated protein kinase (MAPK) phosphorylation were determined by cell based ELISA. Effects of mapracorat on nuclear factor kappa B (NFκB) and activator protein-1 (AP-1) transcriptional activity were assessed by reporter gene assay. Dexamethasone was used as a control.

**Results:**

Hyperosmolar conditions induced release of the pro-inflammatory cytokines interleukin-6 (IL-6), interleukin-8 (IL-8), and monocyte chemotactic protein-1 (MCP-1) from cultured human corneal epithelial cells, and altered the phosphorylation state of p38 and c-Jun N-terminal kinase (JNK) and transcriptional activity of NFκB and AP-1. Incubation of cells with mapracorat inhibited hyperosmolar-induced cytokine release with comparable activity and potency as dexamethasone. This inhibition was reversed by the glucocorticoid receptor antagonist mifepristone (RU-486). Increased phosphorylation of p38 and JNK caused by hyperosmolarity was inhibited by mapracorat. Mapracorat also significantly decreased the hyperosmolar-induced increase in NFκB and AP-1 transcriptional activity.

**Conclusions:**

Mapracorat acts as a potent anti-inflammatory agent in corneal epithelial cells challenged with osmotic stress, with comparable activity to the traditional steroid dexamethasone. These in vitro data suggest that mapracorat may be efficacious in the treatment of dry eye disease.

## Introduction

Dry eye disease is a common pathological condition, involving tear film instability and epithelial damage which leads to symptoms of discomfort and visual disturbances. A central mechanism causing these alterations is an increase in the osmolarity of the tears [1,2]. Tear osmolarity in dry eye patients ranges from 306 to 441 mOsm, with an average of 343 mOsm, compared to ~302 mOsm in non-dry eye patients [3]. However, a recent study suggests that transiently, tear hyperosmolarity may be much higher when local tear film thinning or breakup occur [4]. This increased osmolarity leads to a cycle of inflammation, further exacerbating dry eye symptoms [1,2].

In vitro cell based studies have demonstrated that exposure of the corneal epithelium to hyperosmolarity increases activation of mitogen activated protein kinases (MAPKs) such as c-Jun N-terminal kinase (JNK) and p38, serine/threonine-specific protein kinases that respond to extracellular stimuli and regulate various cellular activities [5,6]. Experimental dry eye models have also demonstrated an increase in MAPKs, especially JNK [7,8]. This leads to increased activity of transcription factors such as activator protein-1 (AP-1) and nuclear factor kappa B (NFκB) which upregulate pro-inflammatory cytokine production in the ocular surface cells leading to an increase in release of cytokines into the tears. Increased levels of multiple cytokines including interleukin-6 (IL-6), interleukin-8 (IL-8), and tumor necrosis factor-alpha (TNF-α) have been detected in both the tear fluid of patients with dry eye symptoms [9-12] and also in animal models of the disease [7,8,13]. These inflammatory mediators then participate in the recruitment of immune cells into the cornea and conjunctiva leading to a continuing cycle of inflammation [14-16].

To date, the only FDA-approved drug for the treatment of dry eye disease in the United States is cyclosporine A (Restasis®) which is purported to act in part by inhibition of T-cell-stimulated cytokines by binding to nuclear proteins required for T-cell activation [17,18]. Topical corticosteroids can be used for anti-inflammatory therapy for amelioration of dry eye syndrome, however their long-term use is limited by the fact that they cause side effects such as increased intraocular pressure (IOP) and cataract formation [17,18]. Side effects of traditional steroids are mostly due to transactivation-dependent gene regulation, whereas their anti-inflammatory properties are mediated largely via transrepression. Recently, selective glucocorticoid receptor agonists (SEGRAs), a new class of anti-inflammatory compounds, have been developed. SEGRAs promote a conformation of the glucocorticoid receptor which preferentially exhibits glucocorticoid-like anti-inflammatory activities, primarily mediated via transrepression. This involves the modulation of the activation and/or activity of transcription factors through altered protein–protein interactions with co-regulatory proteins and promoter-bound transcription factors. SEGRAs exhibit reduced ability to transactivate than steroids and this is thought to be because they exhibit lower glucocorticoid receptor-DNA interactions than steroids. Therefore, these agents may offer a reduced side-effect profile in comparison with classical steroids by dissociation between transactivation and transrepression [19-22].

Mapracorat (also known as BOL-303242-X or ZK 245186) is a novel SEGRA compound under clinical evaluation for the treatment of inflammatory skin and eye diseases. Mapracorat showed anti-inflammatory efficacy similar to traditional glucocorticoids in murine models of skin inflammation and also in multiple ocular cell types [23,24]. It exhibited a better safety profile with regard to growth inhibition and induction of skin atrophy after long-term topical application, thymocyte apoptosis, hyperglycemia, and hepatic tyrosine aminotransferase activity [23]. In trabecular meshwork cells, mapracorat behaved as a partial agonist in contrast to traditional glucocorticoids, in increasing myocilin, a protein thought to be involved in steroid-induced glaucoma [25]. Mapracorat also demonstrated reduced ability to increase IOP in normotensive rabbits when compared to dexamethasone [26].

The ability of mapracorat to reduce inflammation with reduced side effects compared to classical steroids makes it an attractive putative therapeutic for dry eye. Previous studies have demonstrated that hyperosmolarity induces an inflammatory response in cultured human corneal epithelial cells (HCEpiC) [5,27]. Furthermore, the efficacy of several potential dry eye therapeutics has been tested using this model [6,28,29]. Therefore, the current study investigated the anti-inflammatory properties of mapracorat in hyperosmolar-stressed HCEpiC. The effect of mapracorat on hyperosmolar-induced cytokine release was determined in both transformed and primary HCEpiC. To investigate the mechanisms underlying the anti-inflammatory properties of mapracorat, its effects on p38 and JNK MAPKs activation and AP-1 and NFκB transcriptional activity were also investigated.

## Methods

### Reagents

EpiLife medium and human corneal growth supplement (HCGS), penicillin-streptomycin solution, alamarBlue solution, Lipofectamine LTX, and Opti-MEM were obtained from Invitrogen (Carlsbad, CA). Mapracorat (BOL-303242-X, ZK245186; R-1,1,1-trifluoro-4-(5-fluoro-2,3-dihydrobenzofuran-7-yl)4-methyl-2-{[(2-methyl-5-quinolyl)amino]methyl}pentan-2-ol) was provided by Bayer Schering (Berlin, Germany). Dexamethasone was from Sigma (St. Louis, MO) and IL-1β was from R&D Systems (Minneapolis, MN). Human multiplex-cytokine kits were from Millipore (Billerica, MA). Cell-based FACE p38 and JNK ELISA kits were from Active Motif (Carlsbad, CA). Cignal AP1 reporter (luc) and Cignal NFκB reporter (luc) kits were purchased from SA Biosciences (Frederick, MD). Dual-Glo Luciferase assay system was from Promega (Madison, WI). All other reagents were purchased from standard commercial sources and were of the highest available purity.

### Cells and treatments

SV40-transformed human corneal epithelial cells (T-HCEpiC) were received at passage 20 from ATCC (Manassas, VA). Primary HCEpiC (P-HCEpiC; passage 2) were purchased from Invitrogen. Both of these cell types were maintained in EpiLife medium supplemented with HCGS, 100 U/ml of penicillin, and 100 µg/ml of streptomycin at 37 °C in a humidified incubator with 5% CO_2_. Cells were cultured in glucocorticoid-free medium (EpiLife basal medium supplemented with 12.5 µg/ml bovine pituitary extract, 1.25 µg/ml bovine insulin, and 1.25 ng/ml EGF) for 48 h before exposure to hyperosmolar medium. Osmolarity was increased to 440 mOsM by the addition of 123 mM sucrose to basal EpiLife medium (at 317 mOsM). Osmolarity of the solutions was verified using an osmometer (Osmette; Advanced Instruments, Norwood, MA). Dexamethasone and mapracorat stock solutions were prepared in DMSO. Each treatment was performed in at least triplicate, and appropriate dilutions were prepared to deliver a constant amount of the vehicle to each well. Lack of an effect of treatments on cell metabolic activity, an index of cell viability, was determined by the alamarBlue assay [30,31]. A summary of the experiments performed in this study is shown in Table 1, as outlined in detail below.

**Table 1:** Experimental design summary.

** **	**Experiments performed**				
Experiment	Effect of mapracorat on hyperosmolar-induced cytokine release	Effect of mapracorat on hyperosmolar-induced cytokine release	Effect of RU-486 on mapracorat inhibition of hyperosmolar-induced cytokine release	Effect of mapracorat on hyperosmolar-induced MAPK phosphorylation	Effect of mapracorat on hyperosmolar -induced AP-1 and NFκB activities
Technique	Multiplex Luminex	Multiplex Luminex	Multiplex Luminex	Cell-based ELISA	Reporter gene assay
Cell type	T-HCEpiC	P-HCEpiC	T-HCEpiC	T-HCEpiC	T-HCEpiC
Dose range of mapracorat and dexamethasone	1–1000 nM	1–1000 nM	1–1000 nM	0.1–10 µM	0.1–1 µM

### Cytokine release by multiplex Luminex

T-HCEpiC were seeded on 48-well plates at a density of 2×10^4^ cells; P-HCEpiC were seeded on 24-well plates at a density of 1.5×10^4^ cells. After 72 h, cells were treated with vehicle (0.1% DMSO), 10, 30, 100, 300, or 1,000 nM dexamethasone or mapracorat for 24 h in 440 mOsM hyperosmolar basal media. Cytokine content in the culture medium was analyzed using multiplex Luminex bead technology [32,33] according to the manufacturer's instructions. Briefly, 25 µl of medium samples were incubated with antibody-coated capture beads overnight at 4 °C. Washed beads were further incubated with biotin-labeled anti-human cytokine antibodies for 1 h at room temperature followed by incubation with streptavidin-phycoerythrin for 30 min. Samples were analyzed using Luminex 200™ (Luminex, Austin, TX) and Statlia software (Brendan Technologies Inc., Carlsbad, CA). Standard curves of known concentrations of recombinant human cytokines were used to convert median fluorescence intensity (MFI) to cytokine concentration in pg/ml. Only the linear portions of the standard curves were used to quantify cytokine concentrations.

### p38, ERK, and JNK phosphorylation

Cells were seeded on a 96-well plate at a density of 1×10^4^ cells per well. After 48 h, the medium was replaced with basal medium for 18 h. Due to the short incubation time necessary for measurement of MAPK phosphorylation, cells were pre-treated with 0.1-10 µM mapracorat or dexamethasone for 2 h in basal medium. Cells were then treated with isosmolar media, hyperosmolar media, or hyperosmolar media with 0.1-10 µM mapracorat or dexamethasone for 30 min. Levels of phosphorylated and total p38, extracellular signal-related kinase (ERK) or JNK were measured using a cell based ELISA according to the manufacturer’s instructions. Briefly, the cells were fixed with 4% formaldehyde in PBS. After blocking, cells were incubated with primary antibodies specific to the appropriate phosphorylated or total MAPK overnight at 4 °C. Negative controls were performed using secondary antibody only. After washing, cells were incubated with HRP-conjugated secondary antibody for 1 h followed by further washing. Chemiluminescent reagent was added to each well and the resulting luminescence was read on a Synergy plate reader (Biotek, Winooski, VT). To verify that cell number was unchanged by treatments, cells in each well were further stained with crystal violet. The absorbance was read on a Synergy plate reader at 595 nm. Data were expressed as the ratio of phosphorylated to total MAPK, and were normalized by cell number.

### AP-1 and NFκB transcriptional activity

AP-1 and NFκB transcriptional activities were measured using an inducible reporter construct which encoded a firefly luciferase reporter gene under the control of a basal promoter element (TATA box) joined to tandem repeats of specific AP-1 or NFκB transcriptional response elements. This vector was mixed with a constitutively expressing *Renilla* construct (which was used as a normalizer) at a ratio of 40:1. pRSVT-HCEpiC were seeded on a 96-well plate at a density of 1×10^4^ per well. After 24 h, 200 ng of AP-1 or NFκB construct was transfected using Lipofectamine LTX transfection reagent. Thirty h after transfection, cells were treated with vehicle (0.1% DMSO), 440 mOsm media and/or 0.1 or 1 µM mapracorat or dexamethasone for 18 h in basal medium. Firefly and *Renilla* luciferase activity was measured using the Dual-Glo luciferase assay system. Background relative luminescence units (RLU) were subtracted and the ratio of firefly luciferase/*Renilla* luciferase luminescence was then calculated.

### Statistical analysis

Data were expressed as mean±SEM. Statistical analysis was performed using a two-way ANOVA-Contrast test with the statistical software JMP8 (SAS Institute, Cary, NC). P values less than 0.05 were pre-determined to be statistically significant. Concentration versus effect curves for cytokines were plotted using averaged data from all samples. Curve fitting was conducted to estimate the IC_50_ for dexamethasone and mapracorat inhibition of hyperosmolarity-induced IL-6 and IL-8 release. A re-parameterized four-parameter logistic equation was fit to the data using similar methodology as that previously described [34] using JMP8.

## Results

### Mapracorat reduced hyperosmolarity-induced cytokine release with comparable activity to dexamethasone

**Figure 1:**
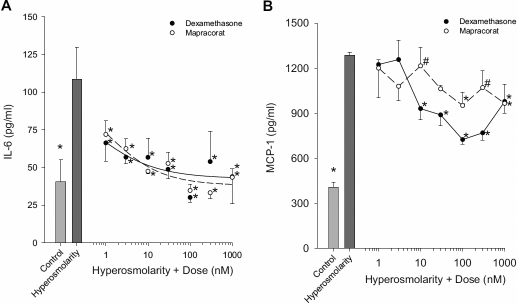
Mapracorat demonstrates similar activity to dexamethasone in inhibiting hyperosmolar-induced cytokine release in T-HCEpiC. Cells were cultured in complete (HCGS containing) medium, followed by glucocorticoid-free medium for 48 h. Cells were then treated with 440 mOsm hyperosmotic basal media in the presence of dexamethasone or mapracorat for 24 h. IL-6 (**A**) and MCP-1 (**B**) release into the media was analyzed by Luminex. Light gray bar represents control (317 mOsm); dark gray bar represents hyperosmolarity (440 mOsm); open circles + dashed line represent mapracorat; closed circles + solid line represents dexamethasone. For **A**, lines are the result of a re-parameterized four-parameter logistic equation fit to the data; for **B**, lines are the linear interpolation between data points. Data are presented as mean±SEM, n=3. *Versus 440 mOsm hyperosmotic media; ^#^versus dexamethasone at the identical dose; p<0.05.

**Figure 2:**
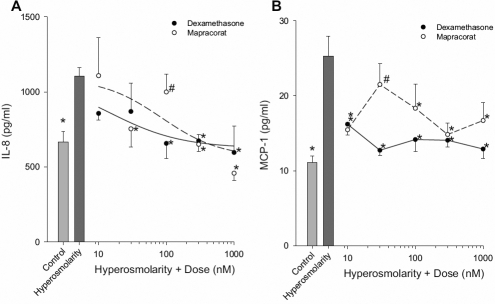
Mapracorat demonstrates similar activity to dexamethasone in inhibiting hyperosmolar-induced cytokine release in P-HCEpiC. Cells were cultured in complete (HCGS containing) medium, followed by glucocorticoid-free medium for 48 h. Cells were then treated with 440 mOsm basal media in the presence of dexamethasone or mapracorat for 24 h. IL-8 (**A**) and MCP-1 (**B**) release into the media was analyzed by Luminex. Light gray bar represents control (317 mOsm); dark gray bar represents hyperosmolarity (440 mOsm); open circles + dashed line represent mapracorat; closed circles + solid line represents dexamethasone. For **A**, lines are the result of a re-parameterized four-parameter logistic equation fit to the data; for **B**, lines are the linear interpolation between data points. Data are presented as mean±SEM, n=3. *Versus 440 mOsm media; ^#^versus dexamethasone at the identical dose; p<0.05.

The effect of hyperosmolarity (440 mOsm) on release of IL-6, IL-8, and MCP-1 in both transformed and primary corneal epithelial cells was determined. IL-6 and MCP-1 release were both significantly increased from T-HCEpiC treated with 440 mOsm hyperosmotic media as compared to 317 mOsm control (Figure 1). There was also a significant increase in IL-8 and MCP-1 levels in the conditioned media from P-HCEpiC exposed to hyperosmolarity (Figure 2).

There was a significant reduction in IL-6 levels measured in the conditioned medium from T-HCEpiC exposed to 440 mOsm hyperosmotic media + all doses of mapracorat and dexamethasone tested (1 – 1,000 nM) as compared to 440 mOsm media alone (Figure 1A). Hyperosmolarity-induced (440 mOsm) increases in MCP-1 levels in the conditioned medium were significantly reduced with 100 and 1,000 nM mapracorat and with 10, 30, 100, 300, and 1,000 nM dexamethasone compared to 440 mOsm media alone (Figure 1B). For the majority of treatments, at the same dose of mapracorat and dexamethasone, there was no significant difference in IL-6 or MCP-1 release from T-HCEpiC. Only at 10 and 300 nM doses of mapracorat, the levels of MCP-1 were significantly higher compared to those at the same doses of dexamethasone. The IC_50_s for dexamethasone and mapracorat inhibition of 440 mOsm hyperosmolarity-induced IL-6 and MCP-1 were at nM levels. Estimated IC_50s_ for IL-6 were 0.97 nM for mapracorat versus 0.38 nM for dexamethasone. The IC_50_ values for dexamethasone and mapracorat inhibition of IL-6 values were not significantly different from each other based on overlapping confidence limits.

**Figure 3:**
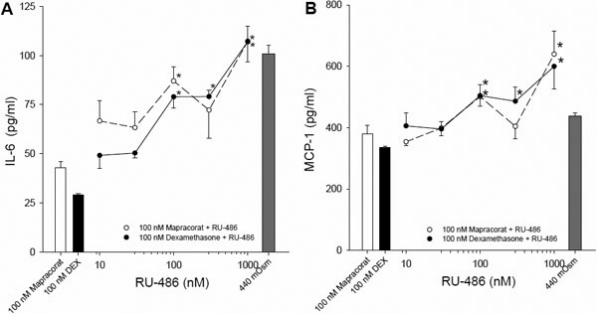
Effect of RU-486 on mapracorat or dexamethasone inhibition of hyperosmolarity-induced cytokine release in T-HCEpiC. Cells were cultured in complete (HCGS containing) medium, followed by glucocorticoid-free medium for 48 h. Cells were treated with 440 mOsm hyperosmotic basal media + RU486 and/or mapracorat or dexamethasone for 24 h. Cytokine release into the media was analyzed by Luminex. **A**: IL-6 release; **B**: MCP-1 release. For both **A** and **B**, the white bar represents 440 mOsm + 100 nM mapracorat and the black bar represents 440 mOsm + 100 nM dexamethasone; open circles + dashed line represents 440 mOsm + mapracorat + RU-486; closed circles + solid line represents 440 mOsm + dexamethasone + RU-486, dark gray bar represents 440 mOsm alone. Data are presented as mean±SEM, n=3. *Versus respective 440 mOsm + mapracorat or 440 mOsm + dexamethasone; for IL-6, dark gray 440 mOsm bar denotes significantly different from 440 mOsm + dexamethasone and 440 mOsm + mapracorat; for MCP-1, dark gray 440 mOsm bar denotes significantly different from 440 mOsm + dexamethasone; p<0.05.

To confirm that the inhibitory effects of mapracorat on cytokine release from HCEpiC were mediated via the glucocorticoid receptor, a widely used glucocorticoid receptor anatagonist, RU-486 (mifepristone), was used. There was an increase in both IL-6 (from 69.9±5.5 pg/ml to 100.8±4.7 pg/ml) and MCP-1 (from 156.3±9.5 pg/ml to 438.2±10.3 pg/ml) levels when cells were exposed to hyperosmolar (440 mOsm) media. There was no effect of 1,000 nM RU-486 alone on 440 mOsm-induced IL-6 (105.5±14.8 pg/ml) or MCP-1 (436.4±9.9 pg/ml) levels. In contrast, IL-6 and MCP-1 levels in the conditioned media were significantly elevated after co-treatment with 100 nM dexamethasone plus RU-468 at 100, 300, and 1,000 nM compared to 440 mOsm/dexamethasone alone (Figure 3). Similarly, co-incubation of 440 mOsm treated cells with 100 nM mapracorat plus either 100 or 1,000 nM RU-486 significantly increased IL-6 and MCP-1 release compared to 440 mOsm/mapracorat alone (Figure 3). This indicates that the anti-inflammatory properties of mapracorat are mediated via the glucocorticoid receptor.

The effect of mapracorat was also determined in primary HCEpiC (P-HCEpiC; Figure 2). There was a dose-dependent inhibition of IL-8 release from P-HCEpiC with significant reduction in hyperosmolarity-induced IL-8 at 30, 300, and 1,000 nM mapracorat and 100, 300 and 1,000 nM dexamethasone (Figure 2A). Both mapracorat and dexamethasone inhibited hyperosmolarity-induced MCP-1 release and a statistically significant effect on MCP-1 was observed at doses as low as 10 nM (Figure 2B). As was the case for T-HCEpiC, for the majority of treatments, at the same dose of mapracorat and dexamethasone, there was no significant difference in IL-8 or MCP-1 release from P-HCEpiC. For IL-6, only at 100 nM mapracorat, levels were higher than at 100 nM dexamethasone and at 30 nM mapracorat, the levels of MCP-1 were higher as compared to 30 nM dexamethasone. The estimated IC_50s_ for inhibition of IL-8 release were estimated as 85 nM for mapracorat and 15 nM for dexamethasone, and, as observed for IL-6 and MCP-1 in T-HCEpiC, were not significantly different based on overlapping confidence limits. For MCP-1 the activities of both compounds were close to maximal at 10 nM, making it difficult to estimate IC_50_ values.

To confirm that these inhibitory effects of mapracorat on cytokine release were not due to an effect on cell viability, an alamarBlue assay was performed. There was no effect of hyperosmolarity or any dose of mapracorat or dexamethasone on cell viability in either cell type.

### Mapracorat and dexamethasone suppress hyperosmolarity-induced phosphorylation/activation of p38 and JNK MAPK

**Figure 4:**
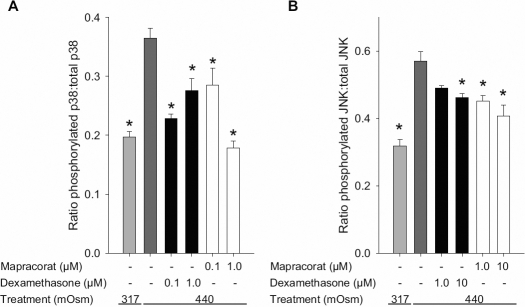
Effects of mapracorat and dexamethasone on hyperosmolarity-induced phosphorylation of p38 and JNK in T-HCEpiC. Cells were cultured in complete (HCGS containing) medium, followed by basal medium for 20 h. Cells were pre-treated with dexamethasone or mapracorat for 2 h. Cells were then treated with 440 mOsm hyperosmotic media + dexamethasone or mapracorat for 30 min. Phosphorylated p38 (**A**) and JNK (**B**) concentrations were determined by cell-based ELISA. Data are presented as mean±SEM, n=6. *Versus 440 mOsm media; p<0.05.

To investigate the underlying mechanisms of the anti-inflammatory effect of mapracorat, its effect on phosphorylation and therefore activation of hyperosmolar-induced stress activated MAPKs were determined using cell-based ELISAs. Exposure of T-HCEpiC to hyperosmolarity (440 mOsM, 30 min) induced an increase in the ratio of phosphorylated to total p38 and JNK, indicating an increase in activity of these inflammatory signaling mediators. In contrast, ERK phosphorylation was slightly decreased after exposure of cells to hyperosmolar medium (ratio of phosphorylated ERK: total ERK, basal=0.87±0.04, hyperosmolarity treated=0.70±0.04, p<0.05). The ratio of phosphorylated to total p38 was inhibited by 0.1 and 1.0 µM of mapracorat and dexamethasone (Figure 4A). Phosphorylated to total JNK ratio was also reduced by 10 µM dexamethasone and both 1 and 10 µM mapracorat (Figure 4B). This indicates that hyperosmolar-induced activities of both p38 and JNK can be inhibited by mapracorat.

### Mapracorat and dexamethasone reduce hyperosmolarity-induced activation of NFκB and AP-1

**Figure 5:**
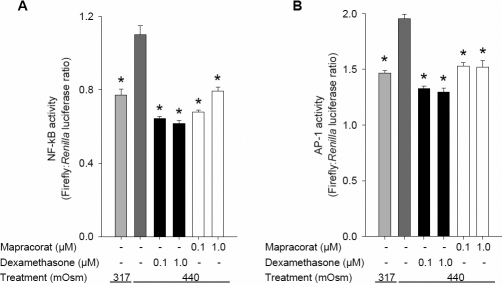
Effects of mapracorat and dexamethasone on hyperosmolarity-induced T-HCEpiC NFκB and AP-1 activity. Cells were transfected with an AP-1 or NFκB reporter gene (expressing firefly) and a constitutively expressing *Renilla* construct. Thirty hours after transfection, cells were treated with 440 media +/− mapracorat or dexamethasone. Cells were assayed for firefly luciferase followed by *Renilla* luciferase luminescence 48 h after transfection. Data are expressed as normalized firefly: *Renilla* luciferase ratio. **A**: NFκB transcriptional activity; **B**: AP-1 transcriptional activity. Data are mean±SEM, n=6. *Versus 440 mOsm media; p<0.05.

Glucocorticoids, through binding to the glucocorticoid receptor, transrepress both AP-1 and NFκB transcription factors [35]. In addition MAPK p38 and JNK regulate production of cytokines through activation of AP-1 [36]. To assess the effects of mapracorat and dexamethasone on the activity of these transcription factors, luciferase reporter gene constructs containing tandem repeats of NFκB or AP-1 specific transcriptional response elements were transiently transfected into T-HCEpiC together with a constitutively expressing *Renilla* construct as a normalizer. Cells were then treated with hyperosmolarity in combination with either mapracorat or dexamethasone. Figure 5 shows that NFκB and AP-1 transcriptional activities were significantly increased by hyperosmolarity (440 mOsM). Mapracorat and dexamethasone at 0.1 or 1.0 µM reduced this hyperosmolarity-induced transcriptional activity to control levels (Figure 5).

## Discussion

Dry eye disease is known to be caused in part by an increase in osmolarity of the tear film resulting in inflammation and subsequent cell damage [1,2]. In both animal and cell based studies, hyperosmotic stress has been shown to induce the expression and production of pro-inflammatory cytokines in the ocular surface cells [5-8]. The key finding of the current study is that mapracorat, a SEGRA compound, inhibited hyperosmolarity-induced pro-inflammatory cytokines IL-6, IL-8, and MCP-1 in HCEpiC, with comparable efficacy and potency as dexamethasone. Two cell types were employed in this study, human primary and human SV40 transformed cells, and the profile of cytokines released by hyperosmolarity differed between the two. While MCP-1 release was induced in both types, IL-6 release was only induced in transformed cells, while IL-8 release was increased only in primary cells. The reason for these differences is unclear, however strain-dependent differences in cytokine release have been observed on the murine ocular surface in response to dessicating stress [13].

IL-6 and IL-8 have been shown to be increased in the tears of dry eye patients in multiple studies [9,10,12]. Inhibition of the receptor for MCP-1, chemokine receptor 2, with a topical agonist significantly improved dry eye disease in a murine model, demonstrating the importance of this cytokine in the pathology of dry eye [37]. We demonstrated that these inhibitory effects of mapracorat on cytokine release were mediated via the glucocorticoid receptor, by reversal using RU-486, a synthetic steroid that antagonizes glucocorticoids such as dexamethasone by competing for the ligand binding site [38]. These data are in agreement with a previous study in HCEpiC which demonstrated that IL-1β-stimulated cytokine release could be inhibited by mapracorat [24].

The MAPK cascades are serine/threonine-specific kinases which play a key role in regulating the production of proinflammatory cytokines leading to inflammation [39]. While JNK and p38 are stimulated by predominantly stress-related stimuli such as osmotic and redox stress and UV radiation, ERK is activated through proliferative and mitogenic stimuli [39]. Previous studies have demonstrated that hyperosmolar-induced cytokine release in HCEpiC is mediated via multiple MAPKs including ERK, JNK, and p38 [5,6]. We found that both JNK and p38 were stimulated by hyperosmolarity and inhibited by mapracorat to a similar extent as dexamethasone. In contrast, we did not detect any increase in ERK phosphorylation in response to hyperosmolarity.

The transcription factors NFκB and AP-1 play a major role in the induction of genes involved in inflammation such as cytokines, cytokine receptors, chemotactic proteins and adhesion molecules [35]. In HCEpiC exposed to hyperosmolarity, p38 is known to regulate NFκB activity [28], while increased JNK activity leads to stimulation of AP-1 activation through activation of Jun [27,36]. In the current study we demonstrated that hyperosmolarity-induced transcriptional activities of AP-1 and NFκB were inhibited by mapracorat. This demonstrates that the anti-inflammatory effects of mapracorat are mediated by effects on multiple signaling pathways.

Like traditional steroids, SEGRAs are glucocorticoid receptor ligands that promote transrepression giving them potent anti-inflammatory properties [40,41]. SEGRAs, like glucocorticoids, transrepress transcription factors such as AP-1 and NFκB by a tethering mechanism or by competition for nuclear co-activators between the GR complex and the transcription factors [40,42,43]. Another mechanism for the reduced AP-1 activity may be through the glucocorticoid receptor binding to JNK leading to suppression of JNK activity and subsequent inhibition of AP-1 [40]. The GC receptor also inhibits the JNK and p38 pathway through increased expression of the MAP kinase phosphatase-1 (MKP-1) which may contribute to the reduced NFκB transcriptional activity [44-46]. This increase in MKP-1 activity is not due to direct binding of the GC receptor to DNA at a GC response element, rather, it is likely mediated by a tethering mechanism involving the glucocorticoid receptor and an enhancer-binding protein which binds to the MKP-1 promoter [47].

SEGRAs exhibit reduced ability to transactivate through glucocorticoid receptor-DNA interactions [40,41]. This confers a major advantage of SEGRAs over traditional glucocorticoids in that they can offer a reduced side effect profile while maintaining anti-inflammatory properties through transrepression. Mapracorat exhibits a better safety profile with regard to growth inhibition and induction of skin atrophy after long-term topical application, thymocyte apoptosis, hyperglycaemia, and hepatic tyrosine aminotransferase activity [23] in animal models. Of importance in its use for ocular indications, mapracorat increases IOP in rabbits to a lower extent than dexamethasone and increases the expression of myocilin, a protein thought to be involved in the progression of glaucoma, to a significantly lesser extent than classic steroids [25,26].

In summary this study demonstrates that a novel SEGRA compound currently under clinical development, mapracorat, decreases hyperosmolarity-induced cytokine release with similar efficacy and potency as dexamethasone. This inhibition is likely mediated by reduction of the activation of MAPKs JNK and p38 and transcriptional activity of AP-1 and NFκB. Given preclinical evidence that mapracorat demonstrates a reduced side-effect profile in the eye, these in vitro results demonstrate the therapeutic potential for mapracorat in the treatment of dry eye disease.
